# Ubiquitin-Specific Protease 29 Exacerbates Cerebral Ischemia-Reperfusion Injury in Mice

**DOI:** 10.1155/2021/6955628

**Published:** 2021-11-16

**Authors:** Jia-Bao Hou, Qian-Ni Shen, Xing Wan, Xu-Ke Liu, Yuan Yu, Mei Li, Wen-Wei Gao, Bo Zhao

**Affiliations:** ^1^Department of Anesthesiology, Renmin Hospital of Wuhan University, Wuhan 430060, China; ^2^Department of Critical Care Medicine, Renmin Hospital of Wuhan University, Wuhan 430060, China

## Abstract

Oxidative stress and apoptosis contribute to the progression of cerebral ischemia/reperfusion (I/R) injury. Ubiquitin-specific protease 29 (USP29) is abundantly expressed in the brain and plays critical roles in regulating oxidative stress and cell apoptosis. The purpose of the present study is to investigate the role and underlying mechanisms of USP29 in cerebral I/R injury. Neuron-specific USP29 knockout mice were generated and subjected to cerebral I/R surgery. For USP29 overexpression, mice were stereotactically injected with the adenoassociated virus serotype 9 vectors carrying USP29 for 4 weeks before cerebral I/R. And primary cortical neurons were isolated and exposed to oxygen glucose deprivation/reperfusion (OGD/R) stimulation to imitate cerebral I/R injury in vitro. USP29 expression was elevated in the brain and primary cortical neurons upon I/R injury. Neuron-specific USP29 knockout significantly diminished, whereas USP29 overexpression aggravated cerebral I/R-induced oxidative stress, apoptosis, and neurological dysfunction in mice. In addition, OGD/R-induced oxidative stress and neuronal apoptosis were also attenuated by USP29 silence but exacerbated by USP29 overexpression in vitro. Mechanistically, neuronal USP29 enhanced p53/miR-34a-mediated silent information regulator 1 downregulation and then promoted the acetylation and suppression of brain and muscle ARNT-like protein, thereby aggravating oxidative stress and apoptosis upon cerebral I/R injury. Our findings for the first time identify that USP29 upregulation during cerebral I/R may contribute to oxidative stress, neuronal apoptosis, and the progression of cerebral I/R injury and that inhibition of USP29 may help to develop novel therapeutic strategies to treat cerebral I/R injury.

## 1. Introduction

Ischemic stroke is a devastating brain attack, and one of the leading causes of death and physical disability worldwide. Currently, the prompt restoration of cerebral blood supply with thrombolytic agents or interventional recanalization is recommended as the first-aid treatment of acute ischemic stroke, which are extremely hampered by the narrow therapeutic time window and secondary injury caused by ischemia/reperfusion (I/R) [1–3]. Mitochondria are the primary source of adenosine triphosphate (ATP) in the brain for energy supply; however, their structure and function are destroyed in the ischemic stage, thereby facilitating reactive oxygen species (ROS) overproduction and oxidative damage after the rapid restoration of cerebral perfusion [4]. And the downregulation of antioxidant enzymes in the brain also accelerates the progression of cerebral I/R injury [5]. In addition, the terminally differentiated neurons are especially sensitive to oxidative stress due to the negligible regenerative capacity and trigger intracellular apoptotic programs in response to excessive free radicals. Therefore, inhibiting oxidative stress and apoptosis are a rational method for the treatment of cerebral I/R injury.

Silent information regulator 1 (SIRT1) belongs to the class III histone deacetylases and confers multiple benefits against oxidative stress and apoptosis [6–8]. And emerging studies have determined a protective role of SIRT1 against cerebral I/R injury. SIRT1-suppressed mice exhibited larger infarct volumes and neurological dysfunction upon ischemic stimulation, while the activation of SIRT1 could significantly suppress I/R-induced oxidative stress, apoptosis, and cerebral injury [9, 10]. In addition to controlling the adaptations to daily environmental changes (e.g., light, temperature, and food availability) for optimal fitness, the circadian clock is also essential for redox homeostasis and cell survival, especially in the brain [11]. The brain and muscle ARNT-like protein (BMAL1) is a core clock gene and is also implicated in the pathogenesis of oxidative damage and cell apoptosis [12]. Our recent study showed that BMAL1 was downregulated in the brain of diabetic mice by I/R injury and that BMAL1 upregulation significantly prevented cerebral I/R injury [13]. Importantly, SIRT1 is reported to deacetylate and upregulate BMAL1, and SIRT1 knockout dramatically decreases BMAL1 expression [14]. Collectively, these findings identify SIRT1 as a promising therapeutic target for cerebral I/R injury.

Ubiquitin-specific proteases (USPs) are a subfamily of deubiquitinases and maintain the stability of target proteins via catalyzing the removal of monoubiquitin or polyubiquitin chains from the substrates [15–17]. Various USPs have been implicated in the pathogenesis of cerebral I/R injury, including USP8, USP14, USP18, and USP22 [18–21]. USP29 belongs to the USP family and is well-known for its role in tumorigenesis [22, 23]. In addition, USP29 also interacted with cyclic GMP-AMP synthase and promoted innate antiviral responses against DNA viruses and autoimmunity [24]. And USP29 is also involved in the regulation of oxidative stress and cell apoptosis. Liu et al. reported that USP29 expression was elevated upon oxidative stress and then promoted cell apoptosis via directly deubiquitinating p53 [25]. Recently, USP29 was found to be abundantly expressed in the brain and contributed to the progression of Parkinson's disease [26]. In the present, we aim to investigate the role and underlying mechanisms of USP29 in cerebral I/R injury.

## 2. Materials and Methods

### 2.1. Reagents

Ex527 (E7034), pifithrin-*α* (PFT-*α*, P4359), 2,3,5-triphenyltetrazolium chloride (TTC, T8877), and lucigenin (M8010) were purchased from Sigma-Aldrich (St. Louis, MO, USA). Enhanced BCA protein assay kit (P0009), 2′,7′-dichlorofluorescin diacetate (DCFH-DA, S0033), total glutathione peroxidase (GPX) assay kit (S0058), Lipo6000™ Transfection Reagent (C0526), and Cell Counting Kit-8 (CCK-8, C0037) were purchased from Beyotime (Shanghai, China). TRIzol™ Reagent (15596018), Amplex™ Red Hydrogen Peroxide/Peroxidase Assay Kit (A22188), and EnzChek Caspase-3 Assay Kit (E13183) were purchased from Invitrogen (Carlsbad, CA, USA). Malondialdehyde (MDA) assay kit (A003), total superoxide dismutase (SOD) assay kit (A001), catalase (CAT) assay kit (A007), and lactate dehydrogenase (LDH) assay kit (A020) were purchased from Nanjing Jiancheng Bioengineering Institute (Nanjing, China). TransAM® NRF2 DNA-binding ELISA Kit (50796) was purchased from Active Motif (Carlsbad, CA, USA), and SIRT1 Activity Assay Kit (ab156065) was purchased from Abcam (Cambridge, UK). The antagomir of microRNA- (miR-) 34a (miR30000542-4-5) and matched control antagomir (miR3N0000002-4-5) were purchased from Guangzhou RiboBio Co., Ltd. (Guangzhou, China). Adenoassociated virus serotype 9 (AAV9) carrying the full length of mouse USP29 or negative control (Ctrl), AAV9 carrying the short hairpin RNA against mouse BMAL1 (shBMAL1) or scramble RNA (shRNA), adenovirus carrying the full length of mouse USP29 (AdUSP29), Cre recombinase (AdCre), or Ctrl (AdCtrl) were generated by Hanbio Biotechnology Co., Ltd. (Shanghai, China). Small interfering RNA against mouse SIRT1 (sc-40987) or BMAL1 (sc-38166) were purchased from Santa Cruz Biotechnology (Dallas, Texas, USA). The primary antibody against USP29 (27522-1-AP) was purchased from Proteintech Group (Chicago, IL, USA). Antiglyceraldehyde-3-phosphate dehydrogenase (GAPDH, 5174), anti-SIRT3 (2627), anti-SIRT1 (8469), anti-BMAL1 (14020), and anti-p53 (2524) were purchased from Cell Signaling Technology (Danvers, MA, USA). And antinuclear factor-E2-related factor 2 (NRF2, ab92946), antiheme oxygenase-1 (HO-1, ab68477), anti-SOD2 (ab68155), anti-B-cell lymphoma-2 (BCL-2, ab194583), anti-BCL-2-associated X protein (BAX, ab182733), and anti-Ac Lys (ab190479) were purchased from Abcam.

### 2.2. Animal Studies

All experimental procedures were performed in compliance with the Guide for the Care and Use of Laboratory Animals by the National Institutes of Health and also approved by the Animal Care and Use Committee of Renmin Hospital of Wuhan University. USP29 floxed (USP29^flox/flox^) mice were generated using CRISPR/Cas9 genome editing system and then bred with CaMKII*α*-Cre mice to generate neuron-specific USP29 knockout (UKO) mice. To establish neuron-specific SIRT1 knockout (SKO) mice, SIRT1 floxed (SIRT1^flox/flox^) mice were bred with CaMKII*α*-Cre mice. Then, UKO and SKO mice were bred with each other to produce neuron-specific USP29/SIRT1 double knockout (DKO) mice [27]. The CaMKII*α*-Cre littermates were selected as the negative controls (CON). All mice were housed in a specific pathogen-free barrier system under constant temperature and humidity with strict 12 h light/dark cycles. The cerebral I/R mouse model was established using a suture embolism as we previously described [13, 28]. Briefly, the male mice aged 8-10 weeks were anesthetized with 5% isoflurane, and then, the left external carotid artery was isolated and exposed, from which the silk plug was inserted into the middle cerebral artery for 1 h. The mice in sham groups received a similar surgery without placing the suture in the middle cerebral artery. Next, all mice were allowed for reperfusion for additional 24 h before being sacrificed. For USP29 overexpression, anesthetized mice were stereotactically injected with the AAV9 vectors carrying USP29 or Ctrl at 2 mm lateral to the bregma and 3 mm under the dura for 4 weeks before cerebral I/R surgery, and the efficiency was validated by western blot [29]. To inhibit SIRT1 in vivo, Ex527 was dissolved in dimethyl sulfoxide (DMSO) and then was intracranially injected into the UKO mice 30 min at a dose of 30 *μ*g before cerebral I/R as previously described [27]. To investigate the potential involvement of BMAL1 in the cerebroprotection of USP29 knockout, UKO mice were anesthetized and stereotactically injected with shBMAL1 or shRNA for 4 weeks before cerebral I/R [29, 30]. In addition, PFT-*α* (1.1 mg/kg/day) and miR-34a antagomir (100 mg/kg/day) were used to inhibit p53 or miR-34a, respectively, according to previous studies [31].

### 2.3. Infarct Volume Measurements

Infarct volume was measured by TTC staining as we previously described [13, 28]. Briefly, the brain was removed and cut into 2 mm coronal sections, which were then immersed in 2% TTC for 30 min at 37°C and fixed in 4% paraformaldehyde overnight. The infarct volume was determined using the ImageJ software in a blinded manner.

### 2.4. Neurological Deficit Score

Neurological deficits were evaluated according to our previous studies based on a neurological grading scale [13].

### 2.5. Brain Water Content Determination

Brain water content was determined to further measure cerebral injury as we previously described [28]. Briefly, the brain was removed and weighed to obtain the wet weight, which was then placed at 105°C for 48 h to obtain the dry weight. The brain water content was calculated using the following formula: (wet weight − dry weight)/wet weight × 100%.

### 2.6. Western Blot

The cerebral samples were homogenized in RIPA lysis buffer, and total protein concentrations were measured by the enhanced BCA Protein Assay Kit according to the manufacturer's instructions [32–34]. Next, 20 *μ*g total proteins were separated by 10% SDS-PAGE and transferred onto PVDF membranes, followed by the incubation with 5% BSA to block the nonspecific binding. And the membranes were then incubated with the primary antibodies at 4°C overnight and stained with HRP-conjugated secondary antibody at room temperature for an additional 1 h. The protein bands were quantified by ImageJ software using GAPDH as the internal control.

### 2.7. Quantitative Real-Time PCR

Quantitative real-time PCR was performed as previously described [35–38]. Briefly, total RNA was extracted using TRIzol™ Reagent and then reversely transcribed to cDNA using a reverse transcription kit. PCR procedures were performed using Bio-Rad CFX Connect Real-Time PCR Detection System (Bio-Rad, USA). GAPDH and U6 were used as the internal control for mRNA and miRNA, respectively.

### 2.8. Assessments of Oxidative Stress

For ROS detection, the brain or cells were lysed and incubated with 50 *μ*mol/L DCFH-DA at 37°C for 30 min protected from the light, which was then measured at the excitation/emission wavelengths of 485/535 nm, respectively, using a Bio-Tek microplate reader. Amplex™ Red Hydrogen Peroxide/Peroxidase Assay Kit was used to detect the levels of cellular hydrogen peroxide (H_2_O_2_) according to the manufacturer's instructions at 560 nm. For superoxide anion (O^2-^) detection, cellular lysates were incubated with 5 mmol/L lucigenin at 37°C for 10 min protected from the light, and the luminescence intensities were measured for 3-5 min at 30 sec intervals. Intracellular MDA content and the activities of total SOD, CAT, and GPX were measured by the commercial kits according to the manufacturer's instructions. To measure NRF2 transcription activity, nuclear extracts were prepared and incubated with the TransAM® NRF2 DNA-binding ELISA Kit according to the manufacturer's instructions, and the absorbance was determined at 450 nm with a reference wavelength of 655 nm on a spectrophotometer.

### 2.9. Determination of Caspase-3 Activity

Caspase-3 activity was determined using Z-DEVD-AMC as a substrate according to the manufacturer's instructions [39]. Briefly, the brain or cells were lysed in the Cell Lysis Buffer and then incubated with the Reaction Buffer and Z-DEVD-AMC substrate at room temperature for 30 min. Finally, the fluorescence was determined with the excitation/emission wavelength at 342/441 nm.

### 2.10. Cell Isolation and Treatments

Primary cortical neurons were isolated from the cortices of E16-E18 mice according to previous studies [29]. Briefly, the cortex was dissected and digested in 0.125% trypsin at 37°C for 10 min, followed by the neutralization in neurobasal medium (GIBCO). Next, the collected suspensions were centrifuged at 1000 rpm for 5 min, and the pellet was resuspended in fresh neurobasal medium containing 10% B27, 0.5 *μ*mol/L glutamine, and 25 *μ*mol/L glutamate, followed by the filtration using a 40 *μ*m strainer. For oxygen glucose deprivation/reperfusion (OGD/R) stimulation, the cells were rinsed with phosphate-buffered saline for 3 times and incubated with glucose-free HBSS buffer at 95% N_2_/5% CO_2_ at 37°C for 4 h and then placed in the fresh neurobasal medium under normal condition for additional 24 h. Neurons cultured in normal oxygen- (95% air, 5% CO_2_) conditioned fresh neurobasal medium for the same periods were used as controls (Ctrl) [40]. For USP29 silence, the primary cortical neurons were isolated from USP29^flox/flox^ mice and then infected with AdCre for 4 h at the multiplicity of infection (MOI) of 20 to knock down the endogenous USP29, followed by the incubation in fresh neurobasal medium for additional 48 h before OGD/R stimulation. For USP29 overexpression, the wild type primary cortical neurons were infected with AdUSP29 at the MOI of 10 for 4 h. To knock down the endogenous SIRT1 or BMAL1, the primary cortical neurons from USP29^flox/flox^ mice were incubated with siSIRT1 (50 nmol/L) or siBMAL1 (50 nmol/L) using Lipo6000™ Transfection Reagent for 4 h and placed in fresh neurobasal medium for additional 24 h before AdCre infection [41, 42]. For p53 inhibition, the wild type primary cortical neurons were pretreated with PFT-*α* (10 *μ*mol/L) for 12 h before OGD/R stimulation [31]. To inhibit miR-34a, the cells were treated with miR-34a antagomir (50 nmol/L) using Lipo6000™ Transfection Reagent for 4 h and kept for additional 24 h before AdUSP29 infection.

### 2.11. Cell Viability and LDH Releases

Cell viability was determined according to previous studies [13, 43]. Briefly, the primary cortical neurons were incubated with 10 *μ*L CCK-8 at 37°C for 4 h and then analyzed at 450 nm using a microplate reader. To measure LDH releases, cell supernatant was collected and measured by the LDH assay kit according to the manufacturer's instructions as we previously described.

### 2.12. Determination of SIRT1 Activity

SIRT1 activity was determined using a fluorometric SIRT1 Activity Assay Kit according to the manufacturer's instructions. Briefly, the brain or primary cortical neurons were lysed under nondenaturing conditions at 4°C and incubated with the Fluorosubstrate Peptide and nicotinamide adenine dinucleotide^+^. Then, the fluorescence intensity was measured with the excitation/emission wavelength at 340/440 nm.

### 2.13. Statistical Analysis

All data are expressed as the mean ± S.D. and analyzed by SPSS software. An unpaired Student's *t* test was performed to compare differences between two groups, while comparisons among three or more groups were determined by the one-way ANOVA analysis followed by Tukey's post hoc test. *P* < 0.05 was considered to be statistically significant.

## 3. Results

### 3.1. USP29 Inhibition Prevents Cerebral I/R Injury in Mice

**Figure 1:**
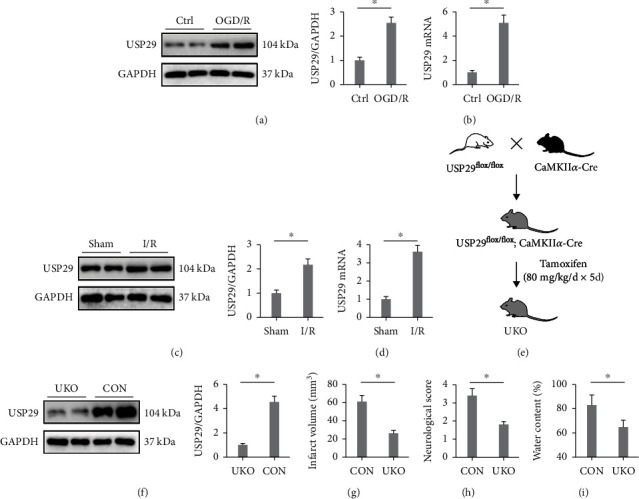
USP29 inhibition prevents cerebral I/R injury in mice. (a, b) Relative expression levels of USP29 protein and mRNA in primary cortical neurons upon OGD/R stimulation. (c, d) Relative expression levels of USP29 protein and mRNA in the brain upon cerebral I/R injury. (e) The generation of neuron-specific USP29 knockout (UKO) mice. (f) Relative protein level of USP29 in UKO mice. (g) Quantification of the infarct volume. (h) The neurological deficit scores. (i) Quantification of brain edema. All data are expressed as the mean ± S.D., *n* = 6 for each group, ^∗^*P* < 0.05.

We first examined the expression of USP29 in the primary cortical neurons upon OGD/R stimulation and found that the protein and mRNA of USP29 were increased in OGD/R-challenged cells (Figures 1(a) and 1(b)). In addition, a similar expression pattern was observed in the brain from I/R mice (Figures 1(c) and 1(d)). To investigate the role of USP29, the neuron-specific USP29 knockout mice were used (Figures 1(e) and 1(f)). As shown in Figure 1(g), UKO mice exhibited a smaller infarct volume upon cerebral I/R injury. And neurological deficit was also improved by USP29 knockout (Figure 1(h)). In addition, USP29 inhibition significantly attenuated cerebral I/R-caused brain edema, as evidenced by the decreased brain water content (Figure 1(i)). The findings enable us to believe that USP29 inhibition prevents cerebral I/R injury in mice.

### 3.2. USP29 Inhibition Diminishes Cerebral I/R-Induced Oxidative Stress and Apoptosis

**Figure 2:**
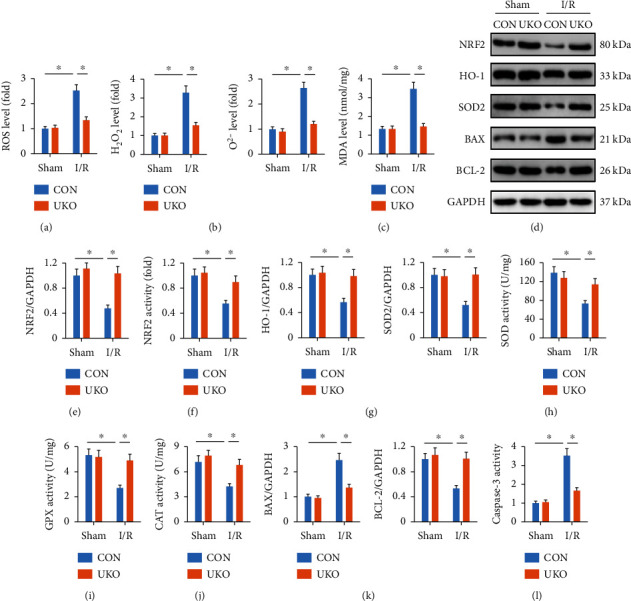
USP29 inhibition diminishes cerebral I/R-induced oxidative stress and apoptosis. (a) Quantification of cerebral ROS by DCFH-DA. (b) Relative levels of cerebral H_2_O_2_ and O^2-^. (c) The levels of MDA production. (d) Representative western blot images. (e, f) Relative levels of NRF2 protein and activity. (g) Relative protein levels of HO-1 and SOD2. (h–j) Quantification of total SOD, GPX, and CAT activities in the brain. (k) Relative protein levels of BAX and BCL-2. (l) Quantification of caspase-3 activity in the brain. All data are expressed as the mean ± S.D., *n* = 6 for each group, ^∗^*P* < 0.05.

Excessive free radicals contribute to the progression of cerebral I/R injury and neuronal apoptosis; therefore, we next measured the role of USP29 inhibition on oxidative stress. As shown in Figures 2(a) and 2(b), ROS generation and the levels of H_2_O_2_ and O^2-^ were decreased in the brain of UKO mice. And USP29 inhibition also inhibited MDA formation, an oxidative product of lipids (Figure 2(c)). NRF2 is a redox-sensitive transcription factor and helps to restrain oxidative stress through inducing the expression of multiple antioxidant enzymes [44, 45]. As shown in Figures 2(d)–2(f), NRF2 expression and activity were significantly inhibited in the brain upon I/R injury but preserved in UKO mice. Accordingly, USP29 knockout also restored the expressions of downstream HO-1 and SOD2 (Figure 2(g)). And the total SOD activity, GPX activity, and CAT activity were also elevated in UKO mice upon cerebral I/R injury (Figures 2(h)–2(j)). ROS overproduction induces oxidative damage to the nonproliferated neurons and provokes cell apoptosis. As shown in Figure 2(k), BAX (a proapoptotic protein) was downregulated, while BCL-2 (an antiapoptotic protein) was upregulated in the brain of UKO mice upon I/R stimulation. And USP29 inhibition also suppressed cerebral I/R-induced caspase-3 activation (Figure 2(l)). These data suggest that knocking down the endogenous USP29 is sufficient to diminish cerebral I/R-induced oxidative stress and apoptosis.

### 3.3. USP29 Overexpression Exacerbates Oxidative Stress, Apoptosis, and Cerebral I/R Injury in Mice

**Figure 3:**
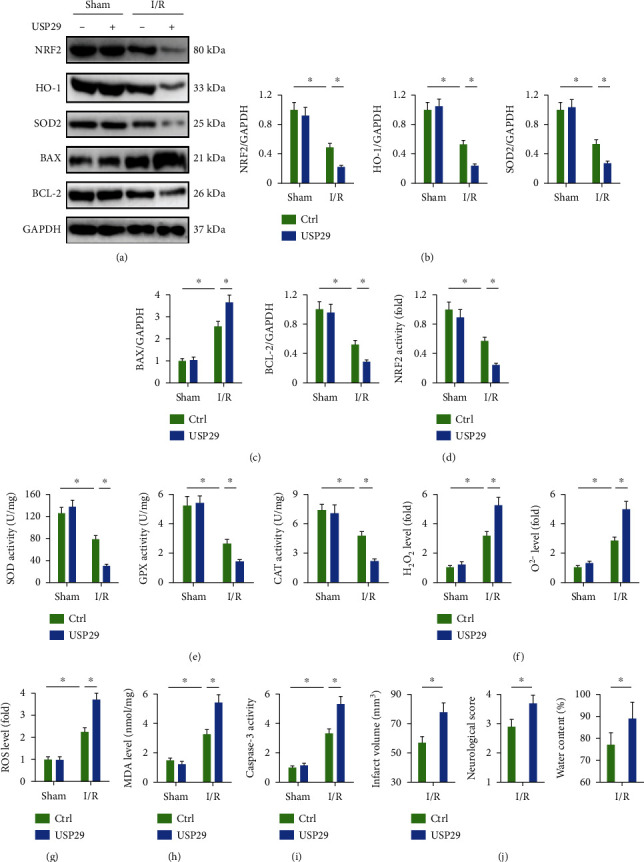
USP29 overexpression exacerbates oxidative stress, apoptosis, and cerebral I/R injury in mice. (a–c) Representative western blot images and the quantification data. (d) Quantification of NRF2 activity in the brain. (e) Quantification of total SOD, GPX, and CAT activities in the brain. (f) Relative levels of cerebral H_2_O_2_ and O^2-^. (g) Quantification of cerebral ROS by DCFH-DA. (h) The levels of MDA production. (i) Quantification of caspase-3 activity in the brain. (j) Quantification of the infarct volume, neurological deficit scores, and brain edema. All data are expressed as the mean ± S.D., *n* = 6 for each group, ^∗^*P* < 0.05.

We next investigated whether enforced USP29 expression would further aggravate cerebral I/R injury in mice. As shown in Figures 3(a) and 3(b), USP29 overexpression further compromised the cerebral antioxidant defenses upon I/R injury, as evidenced by the decreased protein levels of NRF2, HO-1, and SOD2. And BAX expression was further increased, while BCL-2 expression was further decreased in I/R-injured brains with USP29 overexpression (Figure 3(c)). Accordingly, NRF2 activity and the downstream SOD, GPX, and CAT activities were also decreased in USP29-overexpressed brains upon I/R injury (Figures 3(d) and 3(e)). Consistent with the molecular alterations, I/R-induced increases of cerebral ROS, H_2_O_2_, and O^2-^ were augmented by USP29 overexpression (Figures 3(f) and 3(g)). And USP29 overexpression also significantly promoted MDA generation in I/R mice (Figure 3(h)). Caspase-3 is the executor of cell apoptosis, and our data showed that its activity was enhanced in USP29-overexpressed brains upon I/R injury (Figure 3(i)). As expected, USP29 overexpression could exacerbate cerebral I/R injury in mice, as evidenced by the increased infarct volume, neurological deficit score, and brain edema (Figure 3(j)). Based on the above results, we conclude that USP29 upregulation during cerebral I/R injury potentiates oxidative stress, apoptosis, and neurological dysfunction in mice.

### 3.4. USP29 Potentiates I/R-Induced Oxidative Damage and Neuronal Apoptosis In Vitro

Next, we tried to examine the role of USP29 in vitro using the OGD/R model to imitate cerebral I/R injury in vivo. As shown in Figure S1A-B, the survival was decreased, while LDH releases were enhanced in the primary cortical neurons upon OGD/R stimulation, which were attenuated by USP29 silence. We also found that USP29 knockdown significantly suppressed OGD/R-induced increases of caspase-3 activity (Figure S1C). Meanwhile, the levels of ROS, H_2_O_2_, O^2-^, and MDA were decreased in USP29-silenced cells (Figure S1D-E). In contrast, USP29 overexpression further potentiated OGD/R-induced cell death and injury, as evidenced by the decreased cell viability and LDH releases (Figure S1G-H). And the oxidative stress and neuronal apoptosis were also enhanced in USP29-overexpressed neurons upon OGD/R stimulation (Figure S1I-L). The efficiencies of USP29 knockdown and overexpression were validated in Figure S1M. Collectively, these findings imply that USP29 potentiates I/R-induced oxidative damage and neuronal apoptosis in vitro.

### 3.5. USP29 Inhibition Protects against Cerebral I/R Injury through SIRT1 In Vivo and In Vitro

**Figure 4:**
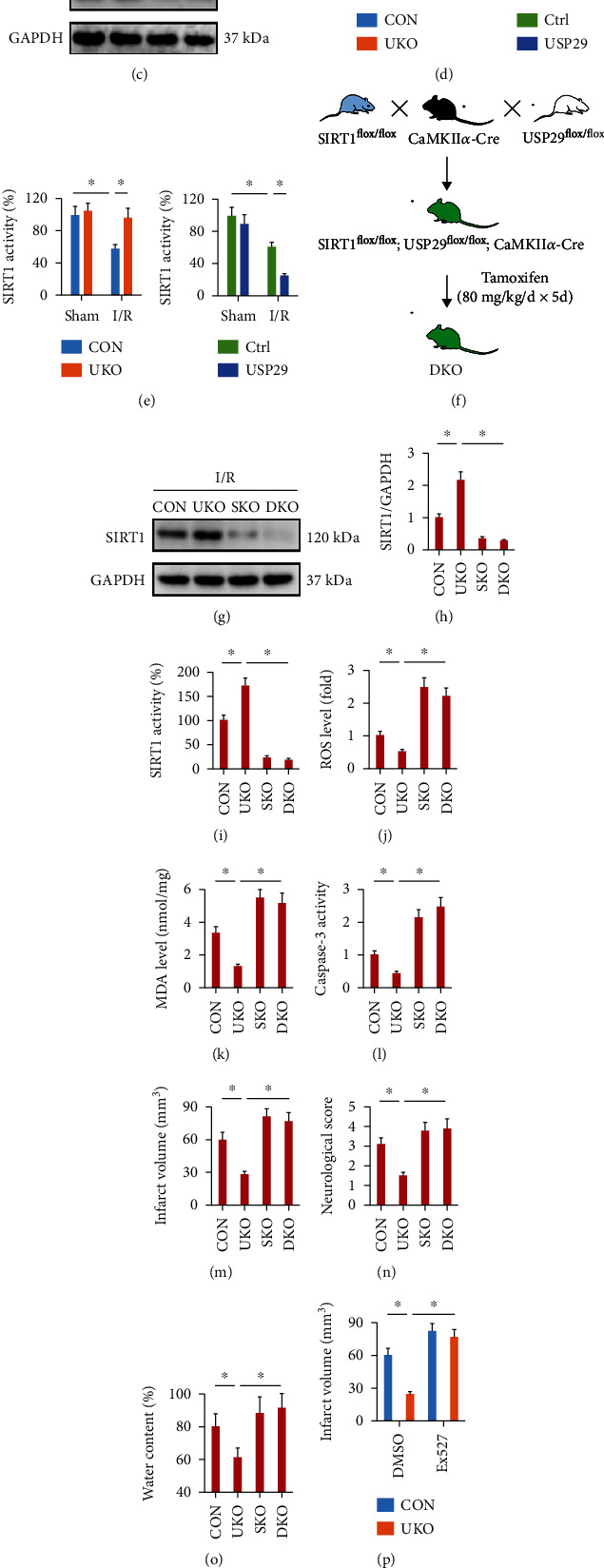
USP29 inhibition protects against cerebral I/R injury through SIRT1 in vivo. (a–d) Representative western blot images and the quantification data. (e) Quantification of SIRT1 activity in the brain. (f) The generation of neuron-specific USP29/SIRT1 double knockout (DKO) mice. (g, h) Representative western blot images and the quantification data. (i) Quantification of SIRT1 activity in the brain. (j) Quantification of cerebral ROS by DCFH-DA. (k) The levels of MDA production. (l) Quantification of caspase-3 activity in the brain. (m) Quantification of the infarct volume. (n) The neurological deficit scores. (o) Quantification of brain edema. (p) The neurological deficit scores. (q) Quantification of brain edema. All data are expressed as the mean ± S.D., *n* = 6 for each group, ^∗^*P* < 0.05.

SIRTs play critical roles in the regulation of oxidative stress and apoptosis and are implicated in the pathogenesis of cerebral I/R injury [9, 10]. Our previous study identified a protective effect of SIRT3 against cerebral I/R injury; therefore, we investigated whether USP29 inhibition prevented cerebral I/R injury through upregulating SIRT3 [28]. As shown in Figures 4(a)–4(d), USP29 inhibition did not affect cerebral SIRT3 expression upon I/R injury but restored cerebral I/R-induced downregulation of SIRT1, while USP29 overexpression further decreased SIRT1 expression in I/R-injured brains. In addition, SIRT1 activity was increased in USP29-suppressed mice but further decreased in USP29-overexpressed mice (Figure 4(e)). To validate whether SIRT1 upregulation is required for USP29 inhibition-mediated cerebroprotection, we generated SIRT1 and USP29 DKO mice (Figures 4(f)–4(i)). As shown in Figures 4(j) and 4(k), the levels of ROS and MDA were decreased in UKO mice upon cerebral IR stimulation, but not in DKO mice. And SIRT1 knockout also blocked USP29 inhibition-mediated inhibition on caspase-3 activity (Figure 4(l)). Accordingly, the improved cerebral I/R injury seen in UKO mice was also abolished in DKO mice, as evidenced by the increased infarct volume, neurological deficit score, and brain edema (Figures 4(m)–4(o)). In addition, we also used a specific inhibitor of SIRT1, Ex527, to further validate the involvement of SIRT1 in these processes. As expected, SIRT1 inhibition by Ex527 completely abolished the cerebroprotection in UKO mice against I/R injury (Figures 4(p) and 4(q)). We also explored the necessity of SIRT1 in USP29 inhibition-mediated cerebroprotection in vitro. As shown in Figure S2A-B, USP29 silence dramatically prevented OGD/R-induced neuronal injury and death, but not in those with siSIRT1 transfection. Accordingly, SIRT1 silence blocked USP29 inhibition-mediated inhibition on caspase-3 activity in OGD/R-stimulated neurons (Figure S2C). And the decreased levels of ROS, H_2_O_2_, O^2-^, and MDA seen in USP29-suppressed neurons upon OGD/R stimulation were elevated in the presence of siSIRT1 (Figure S2D-G). The efficiency of siSIRT1 was presented in Figure S2H. These data suppose that SIRT1 is required for USP29 inhibition-mediated cerebroprotection against I/R injury in vivo and in vitro.

### 3.6. BMAL1 Is Required for SIRT1-Mediated Cerebroprotection in the Context of USP29 Inhibition

**Figure 5:**
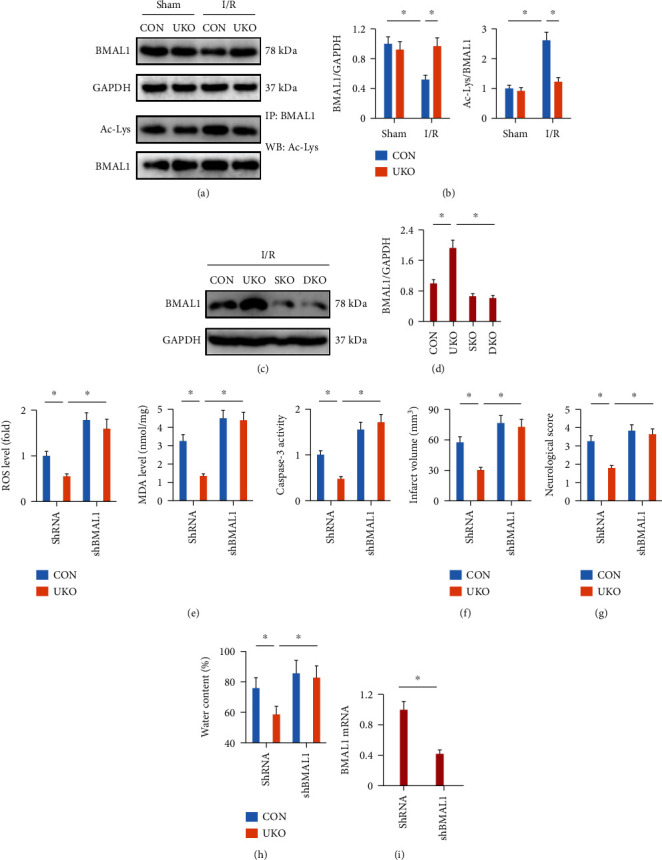
BMAL1 is required for SIRT1-mediated cerebroprotection in the context of USP29 inhibition. (a–d) Representative western blot images and the quantification data. (e) Quantification of cerebral ROS, MDA production, and caspase-3 activity in the brain. (f) Quantification of the infarct volume. (g) The neurological deficit scores. (h) Quantification of brain edema. (i) Relative mRNA level of BMAL1 in the brain with or without shBMAL1 injection. All data are expressed as the mean ± S.D., *n* = 6 for each group, ^∗^*P* < 0.05.

BMAL1, a core regulator in circadian clock, plays critical roles in preventing oxidative damage and cell apoptosis, and our recent study showed that BMAL1 upregulation was sufficient to protect cerebral I/R injury in mice [13]. In addition, SIRT1 is reported to deacetylate and upregulate BMAL1 expression [14]. Therefore, we tried to clarify whether BMAL1 is required for SIRT1-mediated cerebroprotection in USP29-suppressed mice. As shown in Figures 5(a) and 5(b), BMAL1 expression was restored in UKO mice upon cerebral I/R injury, while I/R-induced increase of BMAL1 acetylation was decreased by USP29 inhibition. In addition, SIRT1 knockout significantly blocked USP29 inhibition-mediated upregulation on BMAL1 expression in I/R-injured brains (Figures 5(c) and 5(d)). Next, UKO or CON mice were stereotactically injected with shBMAL1 or shRNA and then received cerebral I/R stimulation. As shown in Figure 5(e), BMAL1 knockdown significantly abolished USP29 inhibition-mediated antioxidant and antiapoptotic effects upon cerebral I/R injury. And the cerebroprotection seen in UKO mice was also abolished after the knockdown of BMAL1, as evidenced by the increased infarct volume, neurological deficit score, and brain edema (Figures 5(f)–5(h)). The efficiency of shBMAL1 was presented in Figure 5(i). In addition, we also investigated the role of BMAL1 in vitro using siBMAL1. As shown in Figure S3A-B, BMAL1 silence significantly abolished the protective effects against OGD/R stimulation in USP29-suppressed neurons, as evidenced by the decreased cell viability and increased LDH releases. And USP29 inhibition-mediated antioxidant and antiapoptotic effects were also blocked in OGD/R-stimulated neurons with siBMAL1 transfection (Figure S3C-H). These findings reveal that BMAL1 is required for SIRT1-mediated cerebroprotection in the context of USP29 inhibition.

### 3.7. USP29 Inhibits SIRT1 Expression via p53/miR-34a Axis

**Figure 6:**
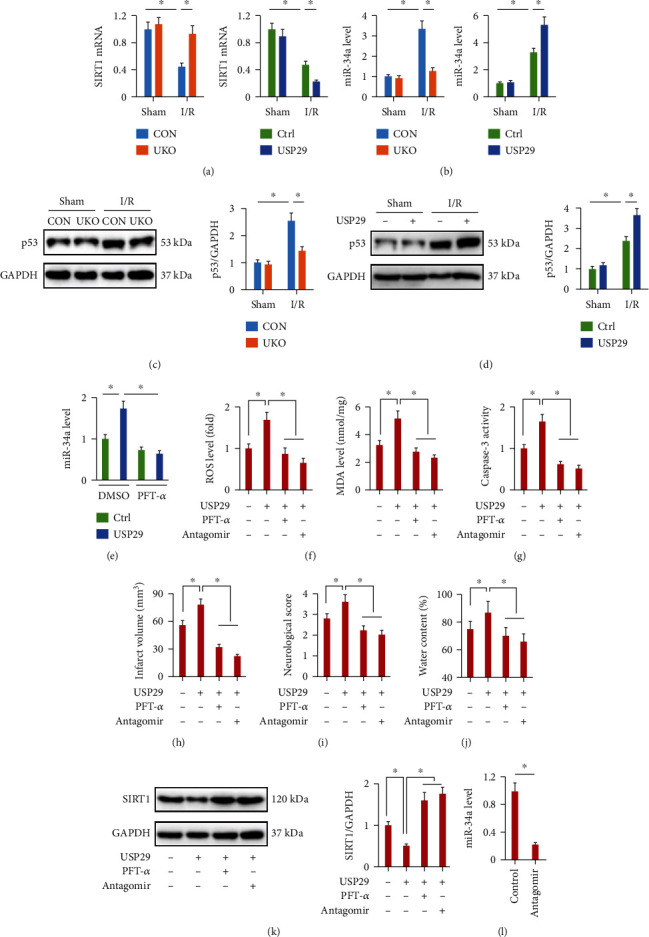
USP29 inhibits SIRT1 expression via p53/miR-34a axis. (a) Relative mRNA level of SIRT1 in the brain. (b) Relative level of miR-34a in the brain. (c, d) Representative western blot images and the quantification data. (e) Relative level of miR-34a in USP29-overexpressed brains with or without PFT-*α* treatment. (f) Quantification of cerebral ROS and MDA production in the brain. (g) Quantification of caspase-3 activity in the brain. (h–j) Quantification of the infarct volume, neurological deficit scores, and brain edema. (k) Relative protein level of SIRT1 in USP29-overexpressed brains treated with PFT-*α* or miR-34a antagomir. (l) Relative level of miR-34a in the brain with or without miR-34a antagomir treatment. All data are expressed as the mean ± S.D., *n* = 6 for each group, ^∗^*P* < 0.05.

Finally, we explored the possible mechanism through which USP29 modulated SIRT1 expression. Consistent with the protein expression, SIRT1 mRNA levels were increased in UKO mice but further decreased in USP29-overexpressed mice upon cerebral I/R injury (Figure 6(a)). In view of the fact that USP29 is a deubiquitinase and modulates the downstream targets via catalyzing the removal of monoubiquitin or polyubiquitin chains at the posttranscriptional level, therefore, we speculated that SIRT1 expression was not directly modulated by USP29. miRNAs are a new class of short noncoding RNAs that negatively modulate gene expression at the posttranscriptional level by binding to the 3′-untranslated regions of target mRNAs, and previous studies by us and the others have identified the indispensable roles of miRNAs in cellular I/R injury [46–48]. miR-34a is an upstream inhibitor of SIRT1 and contributes to the progression of cellular I/R injury through targeting SIRT1 [49, 50]. And our previous study also demonstrated that inhibition of miR-34a significantly prevented hypoxia/reoxygenation-induced cardiomyocyte injury and apoptosis [51]. Therefore, we then detected whether miR-34a was involved in the regulation of SIRT1 by USP29. As shown in Figure 6(b), USP29 inhibition decreased, while USP29 overexpression increased miR-34a level in the brain upon I/R injury. miR-34a is transcriptionally regulated by p53, and USP29 is known to deubiquitinate and stabilize p53 upon oxidative stress [25]. Moreover, we found that p53 expression was decreased in UKO mice and further increased by USP29 overexpression upon cerebral I/R injury (Figures 6(c) and 6(d)). And p53 inhibition by PFT-*α* completely prevented USP29 overexpression-induced upregulation of miR-34a (Figure 6(e)). To further validate the involvement of p53/miR-34a axis in regulating SIRT1 by USP29, USP29-overexpressed mice were pretreated with PFT-*α* or miR-34a antagomir and then received cerebral I/R stimulation. As shown in Figures 6(f) and 6(g), the increased levels of ROS, MDA, and caspase-3 activity seen in USP29-overexpressed mice were suppressed by either PFT-*α* or miR-34a antagomir. And the pretreatment with PFT-*α* or miR-34a antagomir also significantly blocked the deleterious effects of USP29 overexpression upon cerebral I/R injury, as evidenced by the decreased infarct volume, neurological deficit score, and brain edema (Figures 6(h)–6(j)). Accordingly, USP29 overexpression-induced SIRT1 inhibition was prevented in mice treated with either PFT-*α* or miR-34a antagomir (Figure 6(k)). The efficiency of miR-34a antagomir was provided in Figure 6(l). Consistent with the in vivo studies, we observed that either p53 or miR-34a inhibition could attenuate cellular injury and death in AdUSP29-infected neurons upon OGD/R stimulation (Figure S4A-B). Based on these results, we demonstrate that USP29 inhibits SIRT1 expression via the p53/miR-34a axis.

## 4. Discussion

**Figure 7:**
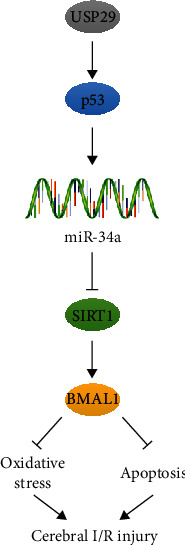
Schematic summary about the role of USP29 in the progression of cerebral I/R injury. USP29 expression is elevated in the brain and primary cortical neurons upon I/R injury, which then inhibits SIRT1/BMAL1 via p53/miR-34a axis to aggravate oxidative stress, apoptosis, and neurological dysfunction.

Despite the in-depth dissections about the mechanisms of cerebral I/R injury, little progress has been made in translating these findings into clinical practice, and the prognosis of these patients remains poor. In the present study, we show that USP29 expression is elevated in the brain and primary cortical neurons upon I/R injury. Neuron-specific USP29 knockout significantly diminishes, whereas USP29 overexpression aggravates cerebral I/R-induced oxidative stress, apoptosis, and neurological dysfunction in vivo and in vitro. Mechanistically, neuronal USP29 enhances p53/miR-34a-mediated SIRT1 downregulation and then promotes the acetylation and suppression of BMAL1, thereby aggravating oxidative stress and apoptosis upon cerebral I/R injury (Figure 7). Generally, our findings for the first time identify that USP29 upregulation during cerebral I/R may contribute to oxidative stress, neuronal apoptosis, and the progression of cerebral I/R injury and that inhibition of USP29 may help to develop novel therapeutic strategies to treat cerebral I/R injury.

Multiple mechanisms are implicated in the pathogenesis of cerebral I/R injury, including oxidative stress and apoptosis. The brain is an organ with high energy utilization and demands for ATP. Mitochondria function as the primary source of intracellular ATP to supply energy for the neurons under physiological conditions; however, they are very susceptible to pathological insults (e.g., hypoxia and toxins) and contribute to the generation of free radicals and cell apoptosis under pathological conditions. Previous studies by us and the others demonstrated that the structure and function of mitochondria were impaired during cerebral I/R injury, accompanied by the increased levels of ROS and neuronal apoptosis [28, 52]. Excessive free radicals also induce peroxidation to biomacromolecules, including lipid, protein, and nucleic acid and aggravate cellular damage and apoptosis. In the present study, we detected increased levels of ROS, H_2_O_2_, O^2-^, and MDA in the I/R-injured brains, and the antioxidant defenses were also compromised upon cerebral I/R injury. SIRT1 is a multifunctional deacetylase and confers cerebroprotection through multiple mechanisms, including the antioxidant and antiapoptotic mechanisms [9, 10]. In the present study, we reported that USP29 inhibition could protect against cerebral I/R-induced oxidative stress and apoptosis through upregulating SIRT1.

Ubiquitination is an important posttranslational modification for intracellular proteins and has pleiotropic biological roles via controlling the ubiquitin-proteasomal degradation. Ubiquitination facilities the rapid turnover of ubiquitinated substrates and cooperates with deubiquitination to regulate the dynamic equilibrium of intracellular proteins [53]. It is well-known that the protein homeostasis is rapidly disrupted by cerebral I/R injury and in turn contributes to the progression of neurological dysfunction [54, 55]. The ubiquitin-proteasome system helps to maintain protein homeostasis in eukaryotic cells through promoting the timely degradation of unnecessary or damaged proteins (e.g., misfolded proteins and oxidized proteins), and it is responsible for the degradation of 80-90% of all intracellular proteins [30, 56]. The ubiquitin-proteasome system-mediated proteolysis involves two important steps: ubiquitination of target proteins and the degradation of the ubiquitinated proteins by the proteasome. Protein ubiquitination is delicately orchestrated by a complex enzymatic reaction, including the covalent attachment of ubiquitin to target proteins by the E1 ubiquitin activating enzyme, E2 ubiquitin conjugating enzyme, E3 ubiquitin ligase, and the removal of polyubiquitin chains by the deubiquitinases. USPs belong to the subfamily of deubiquitinases and are essential for the stability of protein substrates via removing the monoubiquitin or polyubiquitin chains [15, 57]. Some USPs have been implicated in the pathogenesis of cerebral I/R injury. Zhang et al. reported that USP8 downregulation decreased hypoxia-inducible factor 1-alpha protein accumulation in neurons and subsequently enhanced OGD-induced neuron apoptosis [18]. In addition, USP18 overexpression dramatically prevented focal cerebral I/R injury in mice through suppressing microglial activation and inflammation [21]. Conversely, findings from Song et al. indicated that USP14 inhibition could achieve neuroprotective effect upon cerebral I/R injury in vitro and in vivo [19]. Accordingly, USP22 downregulation also attenuated cerebral I/R-induced oxidative stress, inflammation, and cell apoptosis in mice, thereby reducing nerve injury and neurological dysfunction [20]. USP29 is a member of the USP family and functions as an oncogene to promote tumorigenesis in some human tumors. Jo et al. recently detected abundant USP29 expression in the brain and analyzed its role in Parkinson's disease [26]. USP29 expression could be induced by oxidative stress, which in turn promoted cell apoptosis [25]. In contrast, a very recent study by Liu et al. demonstrated that mesenchymal stem cell-derived extracellular vesicles containing USP29 could promote the restoration of traumatic spinal cord injury by stabilizing NRF2 and reducing oxidative stress [58]. In the present study, we observed a significant upregulation of USP29 in the brain and primary cortical neurons upon I/R injury, which then inhibited SIRT1/BMAL1 via the p53/miR-34a axis to aggravate oxidative stress, apoptosis, and neurological dysfunction in vivo and in vitro.

In summary, we prove that USP29 upregulation during cerebral I/R injury is essential for oxidative damage and neuronal apoptosis of the brain through regulating SIRT1 and that targeting USP29 may be helpful for designing effective therapeutic strategies for ischemic stroke.

## Data Availability

The data that support the findings of this study are available from the corresponding author upon reasonable request.

## Supplementary Materials

Supplementary Materials Figure S1: USP29 potentiates I/R-induced oxidative damage and neuronal apoptosis in vitro. Figure S2: USP29 inhibition protects against cerebral I/R injury through SIRT1 in vivo and in vitro. Figure S3: BMAL1 is required for SIRT1-mediated cerebroprotection in the context of USP29 inhibition. Figure S4: USP29 inhibits SIRT1 expression via p53/miR-34a axis.
