# Supplementary material for: Ubiquitin-Specific Protease 29 Exacerbates Cerebral Ischemia-Reperfusion Injury in Mice
Source: Oxid Med Cell Longev. 2021 Nov 16;2021:6955628. doi: 10.1155/2021/6955628 (PMC8610700; doi:10.1155/2021/6955628)
Supplement: Supplementary Materials — Figure S1: USP29 potentiates I/R-induced oxidative damage and neuronal apoptosis in vitro. Figure S2: USP29 inhibition protects against cerebral I/R injury through SIRT1 in vivo and in vitro. Figure S3: BMAL1 is required for SIRT1-mediated cerebroprotection in the context of USP29 inhibition. Figure S4: USP29 inhibits SIRT1 expression via p53/miR-34a axis. [file 6955628.f1.docx]

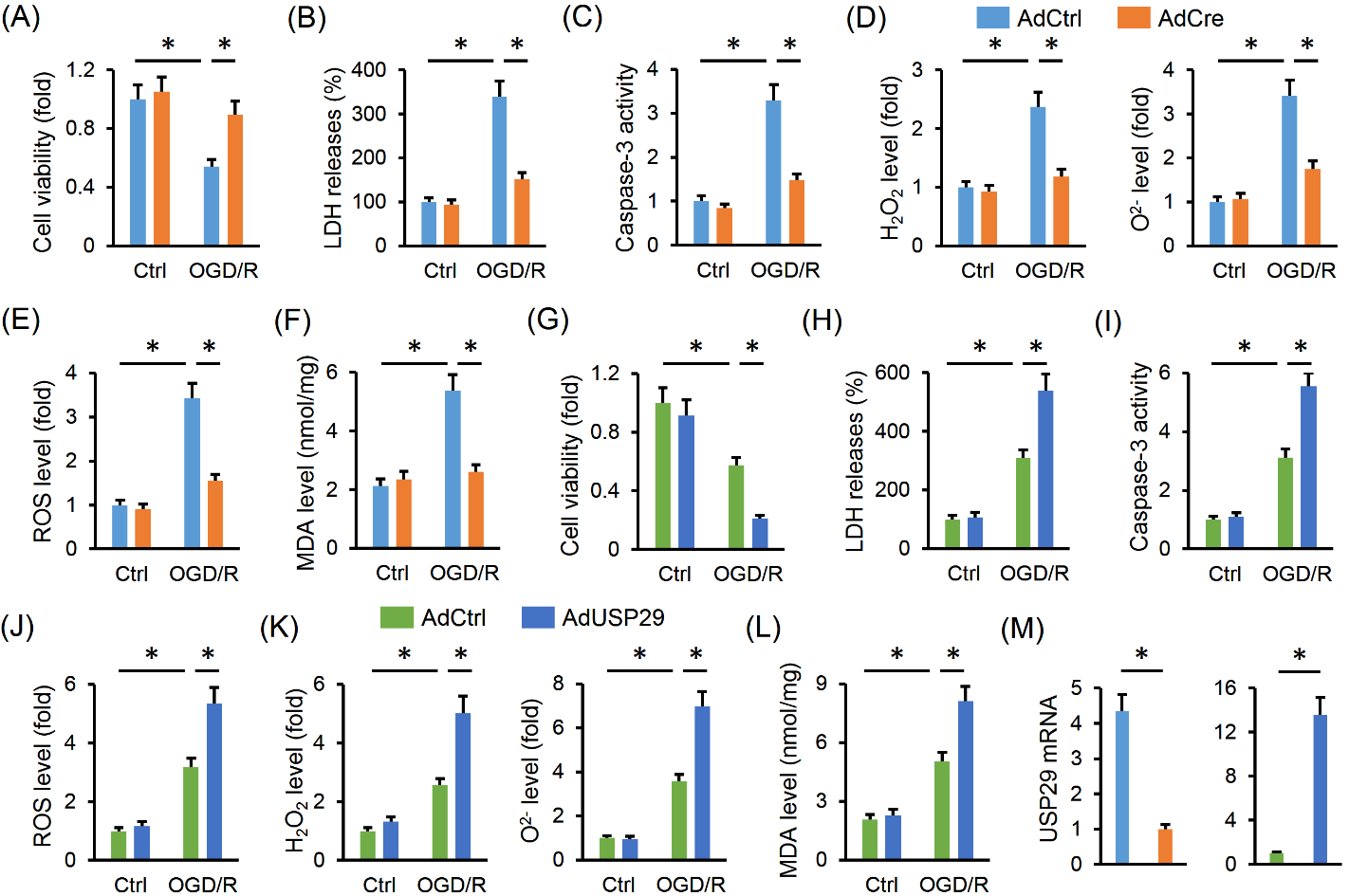


**Figure S1. USP29 potentiates I/R-induced oxidative damage and neuronal apoptosis in vitro.** (A) Quantification of cell viability by CCK-8 assay in USP29-deficient cells. (B) Relative LDH releases to the medium in USP29-deficient cells. (C) Quantification of caspase-3 activity in USP29-deficient primary cortical neurons. (D-F) Relative levels of cerebral H_2_O_2_, O^2-^, ROS and MDA in USP29-deficient cells. (G) Quantification of cell viability by CCK-8 assay in USP29-overexpressed cells. (H) Relative LDH releases to the medium in USP29-oxerexpressed cells. (I) Quantification of caspase-3 activity in USP29-overexpressed primary cortical neurons. (J-L) Relative levels of cerebral H_2_O_2_, O^2-^, ROS and MDA in USP29-overexpressed cells. (M) Relative mRNA levels of USP29-deficient or USP29-overexpressed cells. All data are expressed as the mean ± S.D., n=6 for each group, **P* < 0.05.


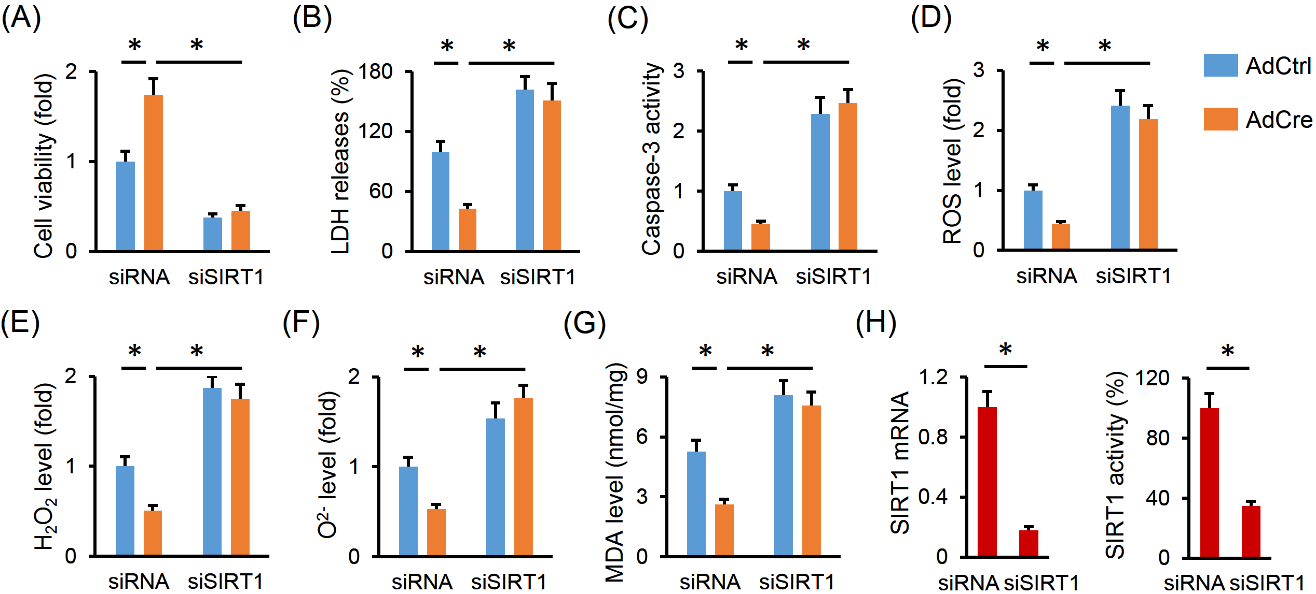


**Figure S2.** **USP29 deficiency protects against cerebral I/R injury through SIRT1 in vivo and in vitro.** (A) Quantification of cell viability by CCK-8 assay. (B) Relative LDH releases to the medium. (C) Quantification of caspase-3 activity. (D-G) Relative levels of cerebral H_2_O_2_, O^2-^, ROS and MDA. (H) Relative levels of SIRT1 mRNA and activity in the primary cortical neurons with or without siSIRT1 transfection. All data are expressed as the mean ± S.D., n=6 for each group, **P* < 0.05.


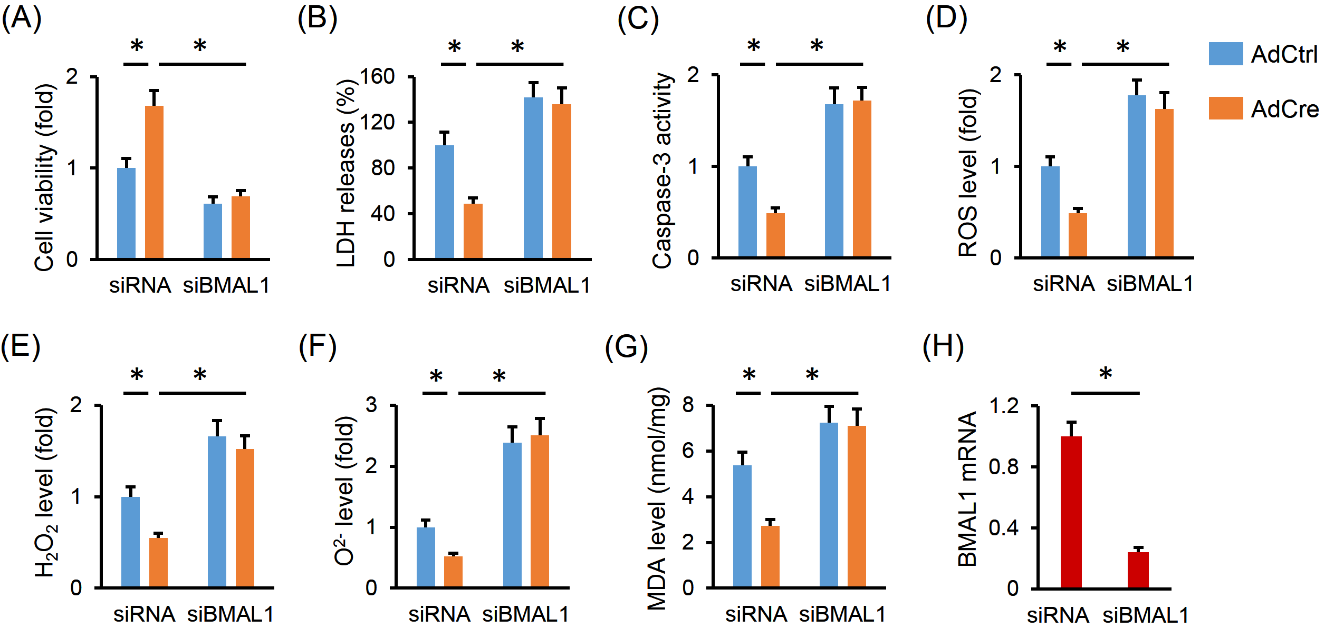


**Figure S3.** **BMAL1 is required for SIRT1-mediated cerebroprotection in the context of USP29 deficiency.** (A) Quantification of cell viability by CCK-8 assay. (B) Relative LDH releases to the medium. (C) Quantification of caspase-3 activity. (D-G) Relative levels of cerebral H_2_O_2_, O^2-^, ROS and MDA. (H) Relative mRNA level of BMAL1 in the primary cortical neurons with or without siBMAL1 transfection. All data are expressed as the mean ± S.D., n=6 for each group, **P* < 0.05.


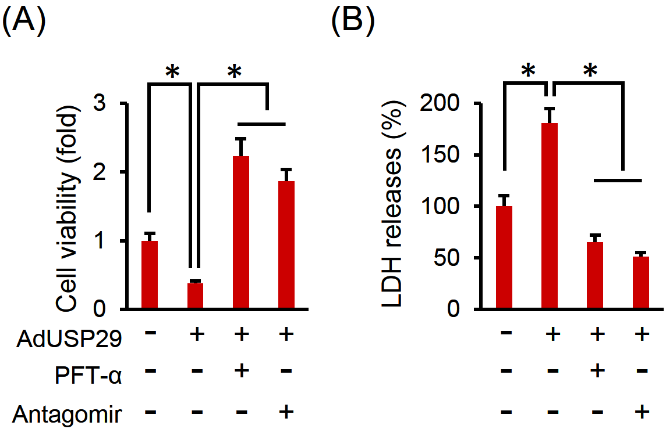


**Figure S4.** **USP29 inhibits SIRT1 expression via p53/miR-34a axis.** (A) Quantification of cell viability by CCK-8 assay. (B) Relative LDH releases to the medium. All data are expressed as the mean ± S.D., n=6 for each group, **P* < 0.05.
